# Plasma enterolactone and risk of prostate cancer in middle-aged Swedish men

**DOI:** 10.1007/s00394-017-1530-z

**Published:** 2017-09-07

**Authors:** Peter Wallström, Isabel Drake, Emily Sonestedt, Bo Gullberg, Anders Bjartell, Håkan Olsson, Herman Adlercreutz, Matti J. Tikkanen, Elisabet Wirfält

**Affiliations:** 1 Nutrition Epidemiology Research Group, Dept. of Clinical Sciences, Lund University, Malmö, Sweden; 2 Clinical Research Centre, Skåne University Hospital, 205 02 Malmö, Sweden; 3 Diabetes and Cardiovascular Disease—Genetic Epidemiology, Dept. of Clinical Sciences, Lund University, Malmö, Sweden; 4 Dept. of Urology, Lund University, Malmö, Sweden; 5 Dept. of Clinical Sciences, Lund University, Malmö, Sweden; 6 Dept. of Cancer Epidemiology, Lund University, Lund, Sweden; 7 Folkhälsan Research Center at University of Helsinki, Haartmaninkatu 8, 00290 Helsinki, Finland; 8 Heart and Lung Center, Helsinki University Hospital, Haartmaninkatu 8, 00290 Helsinki, Finland

**Keywords:** Diet, Prostate cancer, Nested case–control, Lignans, Enterolactone

## Abstract

**Purpose:**

Enterolactone (ENL) is formed in the human gut after consumption of lignans, has estrogenic properties, and has been associated with risk of prostate cancer. We examined the association between plasma ENL levels and prostate cancer in a nested case–control study within the population-based Malmö Diet and Cancer cohort. We also examined the association between plasma ENL and dietary and lifestyle factors.

**Methods:**

The study population consisted of 1010 cases occurring during a mean follow-up of 14.6 years, and 1817 controls matched on age and study entry date. We used national registers (95%) and hospital records (5%) to ascertain cases. Diet was estimated by a modified diet history method. Plasma ENL concentrations were determined by a time-resolved fluoroimmunoassay. Odds ratios were calculated by unconditional logistic regression.

**Results:**

There were no significant associations between plasma ENL and incidence of all prostate cancer (odds ratio 0.99 [95% confidence interval 0.77–1.280] for the highest ENL quintile versus lowest, *p* for trend 0.66). However, in certain subgroups of men, including men with abdominal obesity (*p* for interaction = 0.012), we observed associations between high ENL levels and lower odds of high-risk prostate cancer. Plasma ENL was positively associated with consumption of high-fibre bread, fruit, tea, and coffee; with age, and with height, while it was negatively associated with smoking and waist circumference; however, although significant, all associations were rather weak (*r* ≤ |0.14|).

**Conclusion:**

ENL concentration was not consistently associated with lower prostate cancer risk, although it was weakly associated with a healthy lifestyle.

**Electronic supplementary material:**

The online version of this article (doi:10.1007/s00394-017-1530-z) contains supplementary material, which is available to authorized users.

## Introduction

Estrogen is important to the development and differentiation of the prostate, although our knowledge of the details is incomplete [1]. Lignans constitutes, besides isoflavones (e.g., genistein and daidzein, which are mainly found in soy products) and coumestans (mainly found in sprouts), one of the three main classes of phytoestrogens (PE), non-steroidal plant-based substances with estrogenic properties. In European populations, the lignans is the most important PE class [2]. Lignans are found in plant foods, including flax seeds, fruits, berries, vegetables, and whole-grain foods, which are also important sources of dietary fibre. However, other lignan sources exist, including coffee, tea, fruit juice, and wine [3, 4]. Enterolactone (ENL) is an enterolignan, which in humans is formed in the gut from various dietary lignans through the action of colonic bacteria, and is later detectable in the circulation [5]. ENL has been shown to activate estrogen receptors α and β of the prostate in vivo [6], and may also, for example, modulate the bioavailability of steroidal hormones through competitive binding to sex hormone binding globulin (SHBG) [7]. Several studies have shown protective associations between intake of PE-rich foods, calculated intake of PE and levels of PE in blood or urine, and risk of prostate cancer [8], but most of these findings have been due to isoflavones, which are not generally consumed in large amounts in Europe and North America [8, 9]. International prospective studies have found no association between serum ENL and prostate cancer [10, 11], but a recent meta-analysis by He and Wang et al. [12], including 2 population-based case–control and 4 nested case–control studies, did provide support for a protective association (OR for highest vs. lowest ENL category 0.76, 95% confidence interval 0.60–0.97), although the support from the population-based case–control studies was stronger than that from the nested case–control studies.

In epidemiological studies, ENL concentrations have repeatedly been positively associated with consumption of vegetables, fibre, coffee/caffeine, fruits and berries, whole-grain/high-fibre bread, alcohol/wine and higher education, and negatively associated with recent antibiotic use, smoking, weight/body mass index (BMI), and frequency of defecation [5]; it might thus also be regarded as a potential biomarker of a healthy lifestyle. Furthermore, both body composition (e.g., obesity in general [13] and fat distribution [14]), height [15] and smoking [16] have been implicated as risk factors of prostate cancer.

Thus, the aim of this study was to examine the association between plasma ENL levels and risk of prostate cancer in a population-based cohort. We also aimed to examine associations between diet, lifestyle, and body composition and ENL levels in middle-aged men, since most previous studies on ENL levels have been focused on women, with a few exceptions [17–19].

## Methods

### The Malmö diet and cancer cohort

The Malmö Diet and Cancer (MDC) cohort is a population-based prospective cohort set in the South of Sweden. In 1991, the source population was defined as all persons living in the city of Malmö and born between 1926 and 1945, with Swedish reading and writing skills. In May 1995, the cohort was extended to include all women born between 1923 and 1950 and all men born between 1923 and 1945. With this extension, 74,138 persons constituted the source population. Details of the recruitment procedures and the cohort are described elsewhere [20–23]. With a participation rate of approximately 40%, the cohort consists of 28,098 participants, of whom 11,063 are men.

### Data collection

The study subjects visited the MDC study centre twice (median time between visits: 16 days). At the first visit, dietary interviewers provided information on the background and aim of the project, and detailed instructions about the dietary assessment and the other procedures of the study, including the lifestyle questionnaire. Non-fasting blood samples were drawn from all participants (see below). Anthrompometric measurements and blood pressure were measured by the study nurses. During the second visit, a dietary interview was performed (see below), and the lifestyle questionnaires were checked for incomplete answers.

### Clinical data and case ascertainment

In this study, we excluded men with cancer (except for non-melanoma skin cancers) at baseline (*n* = 485), resulting in a background population of 10,578 men. Incident prostate cancer cases were identified by linkage of personal identification numbers with the Swedish Cancer Register (SCR); the SCR is known to be at least 96% complete [24]. During follow-up until 31st December 2009 (mean 14.6 years), a total of 1016 first-time prostate cancer cases were identified by linkage of the MDC cohort to the SCR.

The controls were sampled from a previous case–control selection from the MDC cohort, consisting of all prostate cancer cases (including prostate cancer both as first-time and subsequent cancer) and three controls per case, matched on age and date of entry into the study [25]. Because of logistic and funding restrictions, we randomly sampled two of the three controls from this selection for each eligible case if controls were free of all cancers (except non-melanoma skin cancers). Due to the more relaxed inclusion criteria of cases and controls in the previous selection, we only included 1828 matched controls in our study.

Data on the clinical and histological characteristics of the tumours were collected from the National Prostate Cancer Register (NPCR), which covers cases from 1996 and onward. Data on cases that occurred between 1991 and 1995 (*n* = 54) were manually extracted from medical records using standard routines. A validation of the NPCR data showed high validity for all variables, including the variables used in the classification of tumours by case severity in this study [26].

High-risk prostate cancer cases (*n* = 356) were defined as local clinical tumour stage T3 or T4, presence of lymph node metastasis (N1), bone metastasis (M1), Gleason score 8 or higher, or serum PSA levels above 50 ng/mL [27]. Tumours were also classified as high-risk if WHO grade was three, and Gleason score was unavailable (*n* = 6). All other tumours were classified as low-risk cases (*n* = 651). Clinical data were insufficient for classification of nine cases as low- or high risk.

Health-conscious men may be prone to attend regular health checks including PSA testing and thus more likely to be diagnosed with prostate cancer at an early stage. This health-conscious behaviour may cause a potential bias when investigating associations between diet and prostate cancer. We, therefore, also investigated symptomatic cases (defined as men who presented with lower urinary tract symptoms or other malignancy-related symptoms) separately (*n* = 469).

### Anthropometric measurements

Anthropometric measurements were taken by trained project staff and obtained from subjects without shoes and wearing light indoor clothing. Body mass index (BMI) was defined as weight (kg) divided by height (m) squared. Waist circumference (cm) was measured midway between the lowest rib margin and iliac crest. Abdominal obesity was defined as a waist circumference of at least 94 cm [28].

### Blood sample handling and analysis

The samples were processed and separated for plasma within 1 h of drawing, as described previously [29]. The samples were stored at −80°C until analysis. The quality control program of the biobank in the MDC study has been described previously [30].

Plasma ENL concentrations were determined by time-resolved fluoroimmunoassay as reported previously [31, 32]. In brief, samples were incubated overnight with a mixture of sulphatase and β-glucuronidase, and unconjugated ENL was extracted with diethyl ether. Sample extracts were then diluted in assay buffer, added with antiserum and europium-labelled and placed in prewashed goat anti-rabbit IgG microstrips to be analysed by Auto-DELFIA 1235 Automatic Immunoassay System. ^3^H-estradiol glucuronide was used as internal standard. All analyses with the automatic instrument were carried out in duplicate. The lower detection limit was 1.5 nmol/L [32]; 227 controls and 130 cases had lower values and were therefore counted as having zero ENL. During the assay development, the intraassay coefficient of variation (CV) varied from 3.3 to 6.0% in the concentrations from 13.2 to 79.5 nmol/L. The interassay CV varied from 6.9 to 9.9% in concentrations from 16.3 to 96.6 nmol/L [32]. The intra-class correlation (ICC) for repeated samples in non-fasting individuals over 5 weeks has been reported to be 0.48 in Malmö [33]. Seventeen measurements failed because of technical reasons, leaving 2827 individuals (1010 cases and 1817 controls) for analysis. The analysing staff was blinded to the case–control status of the samples.

### Dietary assessment

Dietary information was collected using a modified diet history method, combining a 168-item quantitative diet history questionnaire (using exact frequencies and picture booklet to assess portion sizes), a 7-day registration (collecting descriptions of prepared meals, nutrient supplements and cold beverages), and a 45–60 min dietary interview. Energy and nutrient intakes were computed from the total reported food intake using the MDC Food and Nutrient Database, mainly originating from PC Kost2-93 of the Swedish National Food Administration. Data on the validity [34, 35] and reproducibility [36] of the method have been published.

The food and nutrient variables investigated in this study were high-fibre bread, vegetables, fruits and berries, fruit juices, coffee, tea, wine, macronutrients, and dietary fibre. These dietary variables were selected for being sources of dietary lignans and from previous literature on associations between foods and blood ENL levels [17, 19, 37, 38]. Energy percentages of macronutrients were calculated with estimated non-alcohol energy intake, as were fibre intakes per 1000 kcals. The food variables were energy-adjusted by the residual method (ln-transformed data) [39]. A small constant (corresponding to the lowest value of the distribution) was added to all values before ln-transformation. Due to the large number of zero consumers in several food groups, and to skewed intake data, the food group variable distributions were divided into quantiles when used in association tests. Depending on the number of zero consumers (less than 10% or between 10 and 20% of the population), the distributions were divided into either deciles (vegetables, fruit and berries, and coffee) or quintiles (high-fibre bread), respectively. If the proportion of zero consumers was greater than 20%, the zero consumers alone were put into the lowest group, while the rest of the population was divided into quartiles (fruit juice, tea, and wine). Similarly, the ENL distribution was positively skewed and contained a relatively large number of zero measurements; it was therefore divided into quintiles.

For men of the MDC cohort, the relative energy-adjusted validity coefficients (compared to 18 days of weighed foods records) were for dietary fibre 0.74, vegetables 0.65, fruits 0.60, bread 0.50, and wine 0.53 [34].

### Other variables

Information on age was obtained through the Swedish personal identification number, which includes date of birth. A structured questionnaire covering socio-economic and lifestyle factors was completed by the participants. The agreement between the baseline questionnaire and the same questionnaire when repeated after 3 weeks was high for most variables (kappa values >0.75) [22]. Educational level was defined according to the number of years of education completed or degree of educational level attained: elementary, primary and secondary, upper secondary, or university degree (including further education without degree). Smoking was defined as never, former, or current smokers (including irregular smokers). Alcohol habits were classified as none during last month, low (<20 g/day), moderate (20–40 g/day), or high (>40 g/day). Physical activity level was calculated from estimates of average daily total physical activity for each participant [40]. Prevalence of diabetes mellitus (yes/no) was based on information from national and local registries. Current use of *antibiotics* was assessed from self-reported information in the 7-day menu book. Participants were previously classified as potential under-, adequate or over reporters of energy intake [40] using the approach described by Goldberg et al. [41] and later refined by Black [42]. Individuals with potentially unstable dietary habits were identified by the questionnaire item ‘Have you substantially changed your food habits in the past due to illness or other reason?’ [43].

### Statistical methods

The ENL levels, and background characteristics and dietary factors for PCa cases and controls were tabulated; these differences were examined by Kendall’s tau-b, Mann–Whitney and Student’s *t* tests, the latter on ln-transformed data. Most tests involving continuous variables were similarly performed on ln-transformed data to correct for skewed distributions.

We examined the associations between quintiles of plasma ENL among the controls and risk of total, low-risk, high-risk, and symptomatic prostate cancer by unconditional logistic regression, with adjustment for matching variables (age and baseline date), season, educational level, smoking status, height, and waist circumference; the latter variable selected in preference to BMI based on association with plasma ENL (see below) and on previous research [14]. These main associations were further adjusted for consumption of fermented and non-fermented milk and dietary intake of calcium, selenium or zinc. These variables were all selected based on previous scientific literature. Since obesity [13] and smoking [16] have been reported to be associated with PCa mortality, and height with PCa incidence [15], we also examined if the results differed by groups defined by waist circumference (<94/≥94 cm) [28], height (split by median), and smoking status. Due to missing values, 1008 cases and 1817 controls were included in the multivariable analyses.

We tabulated the distributions of anthropometric, other background variables and dietary factors among controls by quintiles of plasma ENL. Associations between the ENL quintiles (categorical) and the continuous other variables were tested with Spearman’s correlation tests. We also listed the distributions of the various background factors by quintiles of plasma ENL among the controls. These associations were tested with Kendall’s tau-b for ordinal variables and *χ* ^2^ tests for nominal variables.

In the next step, the multivariable associations between the selected dietary and body composition variables and plasma ENL deciles among the controls (*n* = 1807) were determined. We first calculated partial correlation coefficients between the individual predictor variables (adjusted for age, baseline date, season, and total energy intake where applicable) and plasma ENL. We also constructed a model in which all food variables were entered simultaneously with the same adjustment as the first model (multivariable food model). Finally, we designed a regression model in which all dietary variables and potential confounders (BMI, waist, height, smoking status, educational status, and physical activity level) were eligible for stepwise (backwards) elimination (elimination criterion was *p* > = 0.10), with adjustment for age, baseline date, and total energy (Full multivariable model). The median values of the food quantiles were used to represent the quantiles in these analyses.

The analyses were repeated with exclusion of individuals who reported dietary change in the past (to include only individuals more likely to have stable food habits and consequently more stable long-term lignan intake and subsequent ENL concentrations). We also repeated the main analyses after exclusion of men with ENL values below the detection limit, and after exclusion of PCa cases occurring within 2 years of blood sampling.

We used the SPSS statistical software version 20 (IBM Corp, U.S.A.) for all analyses, except the calculations of geometric means with confidence intervals, and a post-hoc restricted cubic spline (piecewise) regression on the association between plasma ENL (spline with 3 knots, selected based on Harrell’s recommended percentiles) and risk of high-risk prostate cancer, for which we used Stata version 12.1 (StataCorp LP, U.S.A). The spline was fitted to examine the assumption of a linear association and to identify potential threshold levels in case of non-linearity. The likelihood-ratio test was used to test for linearity of the association by comparing the fit of the linear model to a model that additionally included the cubic spline.

## Results

### Association between ENL and prostate cancer risk

Mean arithmetic plasma ENL among controls and cases were 16.5 (SD 21.0) and 16.3 (SD 16.7) nmol/L, respectively; the corresponding median values were 11.9 and 11.7. There were few differences between cases and controls in terms of unadjusted ENL levels, dietary intakes, and background factors (Tables 1, 2). The most significant difference between cases and controls was the slightly higher proportion of current smokers and low-educated men among controls (Table 1) and the somewhat higher consumption of high-fibre bread among cases (Table 2).

**Table 1:** Background characteristics of cases of prostate cancer and controls in a nested case–control study within the Malmö diet and cancer cohort, 1991–2009

Variable	Cases (*n* = 1010)	Controls (*n* = 1817)	*p* value
Mean (SD)			
Age (years)	60.8 (6.6)	60.6 (6.6)	0.33
Height (cm)	176.6 (6.6)	176.2 (6.4)	0.084
Weight (cm)	81.8 (11.6)	81.5 (11.8)	0.64
BMI (kg/m^2^)	26.2 (3.4)	26.2 (3.4)	0.67
Waist (cm)	93.8 (9.8)	93.7 (9.9)	0.88
WHR	0.94 (0.06)	0.94 (0.06)	0.54
Body fat %	20.6 (4.8)	20.8 (4.9)	0.36
Physical activity level^a^	1.58 (0.35)	1.60 (0.37)	0.24
*N* (%)			
Educational status			0.043
Elementary	445 (44)	848 (47)	
Primary and secondary	182 (18)	368 (20)	
Upper secondary	126 (13)	191 (11)	
Further education/University degree	255 (25)	404 (22)	
Smoking status			0.017
Never-smokers	316 (31)	524 (29)	
Former smokers	468 (46)	800 (44)	
Current smoker	226 (22)	490 (27)	
Alcohol habits			0.50
Zero consumers	67 (7)	134 (7)	
<20 g alcohol per day	666 (66)	1201 (66)	
20–40 g alcohol per day	212 (21)	362 (20)	
>40 g alcohol per day	65 (6)	120 (7)	
Physical activity level			0.66
Quartile 1	251 (25)	455 (25)	
Quartile 2	254 (25)	453 (25)	
Quartile 3	266 (26)	441 (24)	
Quartile 4	239 (24)	468 (26)	
Prevalent diabetes			0.43^b^
Yes	38 (4)	80 (4)	
No	972 (96)	1737 (96)	

Continuous variables: mean (SD) and *t* tests

Categorical variables: numbers (%), Kendall’s tau-b, except where noted

*BMI* body mass index, *WHR* waist–hip ratio

^a^Ratio of total calculated energy expenditure to calculated basal metabolic rate [35]

^b^Fisher’s exact test

**Table 2:** Mean plasma enterolactone and dietary intakes of macronutrients and food sources of lignans in prostate cancer cases and controls of the Malmö Diet and Cancer cohort 1991–2009

Variable	Cases (*n* = 1010)	Controls (*n* = 1817)	*p* value^a^
Plasma enterolactone (nmol/L)	13.4 (12.7–14.2)	13.5 (12.9–14.0)	0.91
Dietary energy (kcals)^b^	2590 (670)	2620 (650)	0.21^c^
Fat (E%)^b^	39.8 (6.3)	39.7 (6.3)	0.65^c^
Carbohydrates (E%)^b^	44.6 (6.0)	44.8 (6.1)	0.45^c^
Protein (E%)^b^	15.6 (2.4)	15.5 (2.5)	0.49^c^
Fibre (g per 1000 kcals)^b^	8.95 (2.5)	8.86 (2.7)	0.37^c^
High-fibre bread (g)	24.3 (22.5–26.2)	23.8 (22.4–25.2)	0.041
Vegetables (g)	147 (142–153)	145 (140–149)	0.34
Fruit and berries (g)	140 (133–147)	135 (130–140)	0.26
Fruit juice (g)	22.5 (18.1–28.0)	26.8 (22.7–31.6)	0.25
Coffee (g)	406 (386–427)	406 (391–421)	0.96
Tea (g)	194 (178–212)	187 (175–199)	0.067
Wine (g)	65.8 (61.0–71.0)	69.1 (65.4–73.0)	0.11

Geometric means (95% confidence intervals), except where noted

*E%* energy percentage

^a^Mann–Whitney tests, except where noted

^b^Arithmetic means (SD)

^c^Student’s *t* tests

We observed no significant associations between plasma ENL and incidence of total, low-risk, high-risk, or symptomatic prostate cancer, although we did note a significantly lower odds ratio for high-risk prostate cancer (0.60; 95% CI 0.40–0.89) in the fourth compared with the first quintile of the ENL distribution (Table 3). Adjustment for consumption of fermented and non-fermented milk or dietary intake of calcium, selenium, or zinc did not change the results (data not shown).

**Table 3:** Odds ratios (with 95% confidence intervals within parentheses) for prostate cancer by quintiles of plasma enterolactone concentration in a nested case–control study of men within the MDC cohort, 1991–2009

Median plasma enterolactone (nmol/L)	Q1	Q2	Q3	Q4	Q5	*P* for trend
	0	6.2	11.9	19.7	36.0	
All prostate cancer (*n* = 1010)						
No. events	201	211	204	181	213	
Model 1^a^	1.00 (ref)	1.01 (0.80–1.30)	0.99 (0.78–1.27)	0.84 (0.66–1.08)	1.06 (0.83–1.36)	0.89
Model 2^b^	1.00 (ref)	1.01 (0.79–1.29)	0.97 (0.76–1.24)	0.80 (0.63–1.04)	0.99 (0.77–1.28)	0.66
Low-risk prostate cancer (*n* = 648)^c^						
No. events	128	131	130	131	128	
Model 1^a^	1.00 (ref)	1.00 (0.75–1.34)	1.01 (0.76–1.35)	0.98 (0.74–1.31)	1.05 (0.79–1.42)	0.73
Model 2^b^	1.00 (ref)	0.98 (0.74–1.31)	0.97 (0.73–1.30)	0.93 (0.69–1.24)	0.97 (0.72–1.31)	0.77
High-risk prostate cancer (*n* = 353)^c^						
No. events	71	77	73	48	84	
Model 1^a^	1.00 (ref)	1.01 (0.71–1.45)	0.96 (0.67–1.38)	0.60 (0.41–0.90)	1.05 (0.73–1.52)	0.82
Model 2^b^	1.00 (ref)	1.01 (0.71–1.46)	0.95 (0.66–1.38)	0.60 (0.40–0.89)	1.02 (0.70–1.47)	0.67
Symptomatic prostate cancer (*n* = 465)^c^						
No. events	87	88	111	83	96	
Model 1^a^	1.00 (ref)	0.97 (0.69–1.35)	1.23 (0.89–1.69)	0.89 (0.63–1.24)	1.08 (0.77–1.51)	0.86
Model 2^b^	1.00 (ref)	0.95 (0.68–1.33)	1.19 (0.86–1.65)	0.84 (0.59–1.18)	0.99 (0.70–1.40)	0.72

Plasma enterolactone was treated as a categorical variable, with median quantile values representing the quantiles in these analyses

*Q* quintile

^a^Unconditional logistic regression model adjusted for age, baseline date, and season

^b^Model additionally adjusted for height (continuous), waist circumference (continuous), educational level (categorical), and smoking status (categorical)

^c^Other cases counted as missing

Because of the lower odds ratio of high-risk prostate cancer in the fourth quintile of the ENL distribution, we wanted to examine whether there was a non-linear association between ENL and OR of high-risk prostate cancer. We thus performed a restricted cubic spline regression (see “Statistical methods”). A model adding the cubic spline to the original linear model did not differ significantly from the original model (likelihood-ratio test, *p* = 0.27). However, a model containing only the spline variable and the covariates revealed that the slopes of the second and third parts of the ENL spline curve were significantly different from the first (*p* = 0.028 and 0.022, respectively), indicating a certain degree of non-linearity (Supplemental Figure 1).

The prostate cancer-ENL associations did not differ by height (<=176/>176 cm, Supplementary Table 1). We noted a significantly inverse association between plasma ENL and risk of high-risk prostate cancer among men with a waist circumference of >94 cm (Q5 vs Q1: OR = 0.65 (0.37–1.12), *p* for trend = 0.024), but not among men with smaller waists (*p* for interaction = 0.012; Supplementary Table 1). No significant trends were noted for the other prostate cancer categories after stratifying by waist circumference. After stratification on smoking status, we observed no significant trends between plasma ENL and risk of any category of prostate cancer, except in current smokers, among whom we noted an inverse association between ENL concentration and risk of symptomatic cancer (*p* = 0.028; Supplementary Table 1). However, this interaction was not statistically significant (*p* for interaction = 0.13).

### Sensitivity analyses

When we excluded men who had reported a substantial change in dietary habits, the ENL associations with prostate cancer were generally weaker. The OR for high-risk cancer in the fourth ENL quintile of high-risk cancer was, however, slightly lower than before [Supplementary Table 2; model 2: OR 0.53 (0.34–0.85), *p* = 0.008]. Furthermore, the associations with prostate cancer were even weaker after removal of individuals with ENL levels below the detection limit. After exclusion of cases occurring within 2 years of sampling, the results were basically unchanged, although a few of the trend tests had their *p* values increased; none of which changed any previously significant results.

### Determinants of ENL concentrations

The highest ENL concentrations were seen in older, taller, and more highly educated men (Supplementary Tables 3, 4). The lowest concentrations were seen among men with central adiposity (large waist circumference), general obesity (BMI), and in current smokers. Furthermore, persons who reported having changed their diet had higher levels than non-changers, and persons who were estimated to have reported their energy intake adequately had higher levels than under reporters of energy. ENL levels differed by season of sampling, with the lowest levels seen during summer (Table 4). We noted, however, that the association with age was mainly due to the considerably higher ENL levels among the oldest age group (70+): mean 31.0 (standard error of the mean [SEM] 3.2) nmol/L among those at least 70 years (*n* = 200), compared to 14.7 (SEM 0.4) nmol/L among the younger men (*t* test for difference *p* < 0.001; data not tabulated).

After adjustment for potential confounders, energy percentage from fat was significantly and negatively associated with plasma ENL levels (*r* = −0.10), while fibre (*r* = 0.19) and carbohydrate (*r* = 0.11) intake were positively associated with ENL (Table 4). Consumption of high-fibre bread, fruit, tea, and vegetables was positively associated with plasma ENL (*r* = 0.06–0.12). Furthermore, BMI, waist circumference, and smoking status were negatively associated with ENL, while height was positively associated.

**Table 4:** Partial correlations between quintiles of plasma enterolactone (dependent) and anthropometric and energy-adjusted dietary variables (independent) among 1817 male controls of a nested case-control study within the Malmö Diet and Cancer cohort (1991-2009)

Variable	Separate models^a^		Multivariable food model^b^		Full multivariable model^c^	
	*r*	*P*	*r*	*P*	*r*	*P*
Energy (kcal)	-0.043	0.067	-0.045	0.067	-0.059	0.012
Fat (E%)^d^	-0.102	<0.001				
Protein (E%)^d^	-0.023	0.32				
Carbohydrates (E%)^d^	0.111	<0.001				
Dietary fibre per 1000 kcal	0.190	<0.001				
High-fibre bread (g)	0.124	<0.001	0.095	<0.001	0.083	<0.001
Vegetables (g)	0.064	0.007	0.019	0.42		NS
Fruit and berries (g)	0.100	<0.001	0.072	0.002	0.071	0.003
Fruit juice (g; 4 cat + zero)	0.028	0.23	0.017	0.46		NS
Coffee (g)	0.022	0.35	0.047	0.047	0.059	0.012
Tea (g; 4 cat + zero)	0.069	0.003	0.061	0.009	0.055	0.019
Wine (g; 4 cat + zero)	0.033	0.15	0.009	0.70		NS
Height (cm)	0.085^e^	<0.001			0.107	<0.001
BMI (kg/m2)	-0.104^e^	<0.001				NS
Waist (cm)	-0.110^e^	<0.001			-0.136	<0.001
Smoking status^f^	-0.120	<0.001			-0.089	<0.001

*r* partial correlation coefficient; *E*% energy percentage; *BMI* body mass index

Food variables expressed as medians of quantiles (deciles, except where noted)

^a^ Adjusted for age, baseline date, season, and total energy (log_e_-transformed)

^b^ As the separate models, but with food variables (quantiles) included simultaneously

^c^ Stepwise backward linear regression model with all food variables (quantiles), BMI (loge-transformed; NS), waist (loge-transformed), height (log_e_-transformed), smoking status, educational status (NS), physical activity level (NS), adjusted for age, baseline date, season and total energy (log_e_-transformed); N.S. is an eliminated variable with *P* > 0.10

^d^ Non-alcohol energy percentage

^e^ Adjusted for age, baseline date, and season

^f^ Never-smokers (category 1), ex-smokers (category 2) and current smokers (category 3)

When the food variables were entered simultaneously (multivariable food model), the associations were slightly weaker, but all previously statistically significant food variables (except vegetables) were still significantly associated with ENL. Furthermore, coffee consumption showed a weakly positive correlation (*r* = 0.047) with ENL (Table 4).

In the full multivariable model, the same food variables (and height) were still significantly positively correlated (although mostly weaker, with *r* values ranging from 0.06 to 0.08) with ENL, while waist circumference, smoking status, and total energy intake were negatively correlated with ENL (Table 4). This model explained 9.9% of the total ENL variation (adjusted *R* ^2^).

### Post-hoc analyses

After noting that plasma ENL levels were clearly higher among men of at least 70 years of age, we examined the associations between ENL and PCa risk in men older and younger than 70 years (Supplementary Table 5). We observed a general tendency to decreased risk at higher ENL values in men who were at least 70 years. For total prostate cancer, the odds ratio of the highest ENL quintile compared to the lowest was 0.53 (95% CI 0.20–1.37, *p* for trend = 0.059, p for interaction = 0.062). For low-risk and high-risk prostate cancers, the associations were somewhat weaker. For symptomatic cancer, however, the OR for the highest quintile was 0.40 (95% C.I. 0.12–1.24, *p* for trend = 0.027, *p* for interaction = 0.023).

## Discussion

There were no significant associations between plasma ENL and incidence of total, low- or high-risk or symptomatic prostate cancer. The results were similar in men above and below median height. However, we noted that higher ENL levels were associated with lower risk of high-risk cancer in men with abdominal obesity and with symptomatic cancer among smoking men. We also noted a similar association between ENL and risk of symptomatic prostate cancer among men at least 70 years. We found ENL in plasma to be associated with consumption of high-fibre bread, fruit and berries, tea, and coffee after adjustment for several potential confounders. It was also positively associated with being at least 70 years of age, and having a greater height. It was negatively associated with smoking and waist circumference.

In general, plasma ENL was not associated with prostate cancer risk in this study. A few, mostly nested case–control studies have reported lower ENL concentrations in prostate cancer cases than in controls [12, 44], with one study also reporting an interaction between an ENL and an SNP in the *MSMB* gene [45]. In contrast, most prospective studies have found no association [10, 11, 46–48]. A recent meta-analysis found a protective association [12], but, again, the latter result was partly driven by data from retrospective case–control studies. An updated meta-analysis [49] noted protective associations between some PE (notably the isoflavones daidzein, genistein, and glyciteine) and PCa, while there was little evidence of effects of dietary lignans and enterolignans such as ENL, although the authors note that their analysis on ENL was hampered by heterogeneity of the included studies [49]. We observed lower risk of symptomatic prostate cancer at higher levels of plasma ENL in older men and in current smokers, and lower risk of high-risk cancer in men with abdominal obesity, although these findings could be spurious. It is noteworthy, however, that these findings concerned high-risk and symptomatic cancers, and were noted among smoking, older, and obese men and groups that have been implicated in aggressive and/or fatal prostate cancer [50, 51].

Our findings on positive associations between plasma ENL and lignan-containing foods and dietary fibre, and negative associations between ENL and smoking and obesity, were generally in agreement with several previous studies [17, 18, 38, 52, 53], including one on women from the same cohort [54]. In general, the correlation coefficients in our study were rather low, but comparable with the previous literature. A recent validation study, evaluating two food frequency questionnaires designed for the estimation of lignan intake, showed a correlation between total lignan intake and serum ENL of 0.22 for one of the FFQ:s and 0.09 for the other [37]. Thus, predicting ENL levels from dietary data is a difficult task. Our study also gives some support to the idea that ENL concentration may be regarded as a marker of a healthy lifestyle, due to the negative associations with smoking and obesity, and positive associations with many foods that contain fibre and lignans [5].

We observed notably higher ENL levels in men older than 70 years. This is a less common finding in the literature. One exception is a Finnish study, in which age was positively associated with ENL levels in women, but not in men. The cause of this finding is unclear. One possibility is confounding by constipation, on which we had no information in this study. Several authors have found constipation be important to the concentration of ENL in plasma [17, 18, 38]. For example: in the study by Kilkkinen et al. [17], constipation was more strongly associated with ENL levels than consumption of either whole grains, vegetables or fruit and berries among men. Similarly, in the Dutch endoscopy population studied by Milder et al., a lower frequency of defecation was as strongly associated with ENL as a hypothesized increase in lignan intake of 3–8 standard deviations [18]. According to a study by Wald et al., the prevalence of constipation among men older than 60 years in the Western world is around 10%, while it is only around 6% in 45–59-year-old men [55]. Thus, a lower defecation frequency among older men might partly explain their higher ENL levels.

Major strengths of our study include the dietary assessment—which is based on a modified diet history method that includes elements of diet recording [56], and produces data of high relative validity—direct body composition measurements and extensive background information, including information on dietary change. Furthermore, this is one of the largest prospective studies on ENL and risk of prostate cancer to date. The loss to follow-up is minimal, and the clinical data are of good quality.

Our study also had some potential weaknesses. The intake of lignans in this population is rather low, at least compared to the other European cohorts participating in the EPIC project [9]. This leads to a comparably narrow intake distribution, which lowers the chance of observing a potential effect. Some Swedish studies have previously reported similarly low ENL concentrations, but at least one Swedish study reported higher concentrations of above 20 nmol/L [19]. In that study, the geometric means for cases were 15.3 nmol/L and those for controls were 21.4 nmol/L, as compared to 13.4 nmol/L for cases and 13.5 nmol/L for controls in our study (Table 2). The cited study was carried out in a different patient population in a different part of Sweden (Stockholm area). Importantly, both studies used the same quantitative, strictly validated method [31, 32]. Thus, the slight differences in ENL concentrations (and the different proportions of participants below the detection limit) most likely reflect different intakes between the populations.

We only had information on antibiotic use during the diet registration period of 7 days; furthermore, most participants had their blood drawn several days before the registration. It has been shown that ENL levels may be significantly lower in antibiotic users up to 16 months after use, presumably through an effect on colonic microbiota [57]. This might obscure any associations between ENL levels and foods or risk of prostate cancer.

We also had only one measurement of plasma ENL. Although the intra-class correlation coefficient (ICC) has been reported to be fair (0.44) for a period of up to 3 years [58], the blood in our study was drawn from non-fasting persons. We have previously estimated the ICC for repeated non-fasting samples during a 5-week period to be 0.48 (95% CI, 0.22–0.72) in women of the MDC cohort [33], which certainly could deflate the measured associations.

We lack information on prostate cancer among close relatives of the participants. Since family history is one of the few established risk factors of prostate cancer, this lowers the precision of our estimates. However, family history is unlikely to be a major influence on ENL levels, and is thus probably not a confounder in this context.

As always, the risk of residual confounding and of chance findings, particularly in studies performing large numbers of statistical tests, must always be considered.

To conclude, this population-based prospective study showed no consistent associations between high levels of plasma ENL and subsequent risk of prostate cancer, which is in line with other prospective studies. We did, however, observe lower risks of high risk or symptomatic cancer at higher ENL levels in men with central adiposity, current smokers, and men who were at least 70 years, respectively. The latter findings are suggestive, but would need to be confirmed in other studies. The study also showed that plasma ENL is a weak marker of consumption of foods such as fruit and berries, tea, and coffee in middle-aged Swedish men. It might possibly be regarded as a marker of a healthy lifestyle, since it was also associated with lower likelihood of smoking and obesity.

## Electronic supplementary material

Below is the link to the electronic supplementary material.

Supplementary material 1 (DOCX 35 kb)

Supplementary material 2 (DOC 31 kb)
