# Supplementary material for: Plasma enterolactone and risk of prostate cancer in middle-aged Swedish men
Source: Eur J Nutr. 2017 Sep 7;57(7):2595–606. doi: 10.1007/s00394-017-1530-z (PMC6182673; doi:10.1007/s00394-017-1530-z)
Supplement: Supplementary file 1 — Supplementary material 1 (DOCX 35 kb) [file 394_2017_1530_MOESM1_ESM.docx]

**Supplementary Table 1** Odds ratios (with 95 % confidence intervals within parentheses) for prostate cancer by quintiles of plasma enterolactone concentration in men by waist circumference above and below 94 centimetre, by smoking status, and by height above and below 176 centimetres in a nested case-control study within the Malmö Diet and Cancer cohort, 1991-2009

|  | | **Waist circumference** | | | **Smoking status** | | | |
| --- | --- | --- | --- | --- | --- | --- | --- | --- |
|  | **No. events** | **<94 cm (n=1472)** | **≥94 cm (n=1343)** | **No. events** | | **Never-smokers (n=834)** | **Ex-smokers (n=1267)** | **Current smokers (n=714)** |
| Total prostate cancer |  |  |  |  | |  |  |  |
| Q1 | 83/118 | 1.00 (ref) | 1.00 (ref) | 55/90/56 | | 1.00 (ref) | 1.00 (ref) | 1.00 (ref) |
| Q2 | 102/109 | 1.03 (0.72-1.48) | 0.98 (0.70-1.38) | 62/92/57 | | 1.03 (0.64-1.67) | 0.98 (0.68-1.42) | 1.01 (0.64-1.59) |
| Q3 | 105/99 | 1.05 (0.73-1.51) | 0.87 (0.62-1.23) | 52/98/54 | | 0.65 (0.40-1.04) | 1.13 (0.78-1.63) | 1.12 (0.69-1.80) |
| Q4 | 95/85 | 0.80 (0.55-1.15) | 0.82 (0.57-1.17) | 65/85/30 | | 0.78 (0.49-1.26) | 0.87 (0.60-1.26) | 0.67 (0.39-1.14) |
| Q5 | 127/85 | 1.13 (0.79-1.61) | 0.85 (0.59-1.23) | 80/123/29 | | 0.99 (0.62-1.59) | 1.04 (0.72-1.50) | 0.84 (0.48-1.48) |
| *P* for trend |  | 0.70 | 0.28 |  | | 0.89 | 0.98 | 0.26 |
| *P* for interaction |  | 0.39 | |  | | 0.59 | | |
| Low-risk prostate cancer |  |  |  |  | |  |  |  |
| Q1 | 58/70 | 1.00 (ref) | 1.00 (ref) | 38/57/33 | | 1.00 (ref) | 1.00 (ref) | 1.00 (ref) |
| Q2 | 65/66 | 0.94 (0.62-1.43) | 1.01 (0.67-1.50) | 43/57/31 | | 0.99 (0.57-1.70) | 0.97 (0.63-1.49) | 0.99 (0.56-1.75) |
| Q3 | 70/60 | 1.00 (0.66-1.52) | 0.89 (0.59-1.35) | 29/67/34 | | 0.50 (0.28-0.88) | 1.24 (0.81-1.90) | 1.29 (0.73-2.28) |
| Q4 | 63/67 | 0.76 (0.50-1.15) | 1.12 (0.75-1.68) | 43/63/24 | | 0.71 (0.41-1.23) | 1.03 (0.67-1.58) | 0.98 (0.53-1.81) |
| Q5 | 74/54 | 0.94 (0.62-1.43) | 0.97 (0.62-1.50) | 50/66/12 | | 0.85 (0.50-1.46) | 1.11 (0.72-1.71) | 0.64 (0.30-1.35) |
| *P* for trend |  | 0.63 | 0.96 |  | | 0.72 | 0.67 | 0.27 |
| *P* for interaction |  | 0.58 | |  | | 0.77 | | |
| High-risk prostate cancer |  |  |  |  | |  |  |  |
| Q1 | 25/46 | 1.00 (ref) | 1.00 (ref) | 17/31/23 | | 1.00 (ref) | 1.00 (ref) | 1.00 (ref) |
| Q2 | 34/43 | 1.13 (0.64-1.99) | 0.96 (0.60-1.55) | 18/33/26 | | 1.09 (0.51-2.30) | 0.99 (0.57-1.70) | 0.98 (0.52-1.87) |
| Q3 | 35/38 | 1.16 (0.66-2.04) | 0.83 (0.51-1.36) | 23/30/20 | | 0.98 (0.49-1.97) | 0.96 (0.54-1.69) | 0.90 (0.45-1.79) |
| Q4 | 30/18 | 0.87 (0.48-1.55) | 0.41 (0.23-0.75) | 22/20/6 | | 0.97 (0.45-1.96) | 0.56 (0.30-1.04) | 0.30 (0.11-0.78) |
| Q5 | 53/30 | 1.57 (0.91-2.69) | 0.65 (0.37-1.12) | 30/36/17 | | 1.31 (0.65-2.66) | 0.92 (0.53-1.61) | 1.04 (0.50-2.19) |
| *P* for trend |  | 0.105 | 0.024 |  | | 0.42 | 0.51 | 0.54 |
| *P* for interaction |  | 0.012 | |  | | 0.56 | | |
| Symptomatic prostate cancer |  |  |  |  | |  |  |  |
| Q1 | 36/51 | 1.00 (Pref) | 1.00 (ref) | 22/40/25 | | 1.00 (ref) | 1.00 (ref) | 1.00 (ref) |
| Q2 | 40/48 | 0.92 (0.56-1.52) | 0.98 (0.62-1.54) | 30/33/25 | | 1.26 (0.66-2.39) | 0.77 (0.46-1.29) | 0.93 (0.50-1.75) |
| Q3 | 59/52 | 1.32 (0.83-2.12) | 1.07 (0.68-1.67) | 30/48/33 | | 0.94 (0.50-1.75) | 1.24 (0.76-2.01) | 1.44 (0.78-2.68) |
| Q4 | 41/41 | 0.78 (0.47-1.28) | 0.90 (0.56-1.45) | 32/41/9 | | 0.99 (0.53-1.86) | 0.95 (0.58-1.56) | 0.43 (0.19-0.98) |
| Q5 | 61/34 | 1.21 (0.75-1.96) | 0.75 (0.45-1.26) | 40/47/8 | | 1.26 (0.68-2.35) | 1.04 (0.64-1.71) | 0.48 (0.20-1.18) |
| *P* for trend |  | 0.52 | 0.23 |  | | 0.56 | 0.68 | 0.028 |
| *P* for interaction |  | 0.22 | |  | | 0.13 | | |

Unconditional logistic regression; models adjusted for age, baseline date, height, waist circumference, education, smoking habits and season.

Abbreviation: Q, quintile.

**Supplementary Table 1, continued**

|  |  | **Height** | |  |
| --- | --- | --- | --- | --- |
|  | **No. events** | **<=176 cm (n=1438)** | **≥176 cm (n=1377)** |  |
| Total prostate cancer |  |  |  |  |
| Q1 | 111/90 | 1.00 (ref) | 1.00 (ref) |  |
| Q2 | 113/98 | 1.11 (0.79-1.55) | 0.92 (0.64-1.33) |  |
| Q3 | 93/111 | 0.86 (0.61-1.22) | 1.09 (0.77-1.56) |  |
| Q4 | 86/94 | 0.71 (0.50-1.00) | 0.93 (0.64-1.34) |  |
| Q5 | 102/110 | 1.02 (0.71-1.44) | 0.98 (0.68-1.43) |  |
| *P* for trend |  | 0.60 | 0.95 |  |
| *P* for interaction |  | 0.62 | |  |
| Low-risk prostate cancer |  |  |  |  |
| Q1 | 74/54 | 1.00 (ref) | 1.00 (ref) |  |
| Q2 | 66/65 | 0.97 (0.65-1.43) | 1.05 (0.68-1.60) |  |
| Q3 | 57/73 | 0.79 (0.52-1.20) | 1.23 (0.81-1.87) |  |
| Q4 | 64/66 | 0.80 (0.54-1.20) | 1.11 (0.72-1.71) |  |
| Q5 | 61/67 | 0.93 (0.62-1.41) | 1.04 (0.67-1.62) |  |
| *P* for trend |  | 0.68 | 0.97 |  |
| *P* for interaction |  | 0.55 | |  |
| High-risk prostate cancer |  |  |  |  |
| Q1 | 36/35 | 1.00 (ref) | 1.00 (ref) |  |
| Q2 | 46/31 | 1.38 (0.84-2.25) | 0.73 (0.43-1.26) |  |
| Q3 | 36/37 | 1.00 (0.60-1.68) | 0.90 (0.53-1.52) |  |
| Q4 | 21/27 | 0.52 (0.29-0.94) | 0.68 (0.38-1.19) |  |
| Q5 | 40/43 | 1.12 (0.67-1.99) | 0.93 (0.54-1.58) |  |
| *P* for trend |  | 0.56 | 0.98 |  |
| *P* for interaction |  | 0.75 | |  |
| Symptomatic prostate cancer |  |  |  |  |
| Q1 | 47/40 | 1.00 (ref) | 1.00 (ref) |  |
| Q2 | 50/38 | 1.17 (0.74-1.85) | 0.78 (0.47-1.28) |  |
| Q3 | 57/54 | 1.26 (0.81-1.97) | 1.14 (0.71-1.83) |  |
| Q4 | 32/50 | 0.64 (0.39-1.05) | 1.05 (0.65-1.70) |  |
| Q5 | 50/45 | 1.21 (0.75-1.93) | 0.81 (0.49-1.35) |  |
| *P* for trend |  | 0.96 | 0.62 |  |
| *P* for interaction |  | 0.94 | |  |

**Supplementary Table 2** Sensitivity analysis: Odds ratios (with 95 % confidence intervals within parentheses) for prostate cancer by quintiles of plasma enterolactone concentration in a nested case-control study of men within the MDC cohort, 1991-2009^[[1]](#footnote-1)^

|  |  | **Q1** | **Q2** | **Q3** | **Q4** | **Q5** | ***P* for trend** |
| --- | --- | --- | --- | --- | --- | --- | --- |
| **All prostate cancer** | **Model 1^[[2]](#footnote-2)^** | 1.00 (ref) | 0.98 (0.75-1.28) | 0.92 (0.70-1.22) | 0.83 (0.63-1.11) | 1.00 (0.75-1.34) | 0.89 |
|  | **Model 2^[[3]](#footnote-3)^** | 1.00 (ref) | 1.04 (0.74-1.46) | 1.00 (0.71-1.40) | 0.83 (0.59-1.17) | 1.03 (0.73-1.45) | 0.87 |
|  | **Model 3^[[4]](#footnote-4)^** | 1.00 (ref) | 1.03 (0.80-1.32) | 0.97 (0.75-1.25) | 0.80 (0.62-1.04) | 1.02 (0.79-1.32) | 0.77 |
| **Low-risk prostate cancer** | **Model 1** | 1.00 (ref) | 1.00 (0.72-1.37) | 0.94 (0.68-1.30) | 1.04 (0.75-1.44) | 0.99 (0.70-1.39) | 0.97 |
|  | **Model 2** | 1.00 (ref) | 0.87 (0.59-1.26) | 0.85 (0.58-1.25) | 0.82 (0.56-1.20) | 0.86 (0.58-1.26) | 0.63 |
|  | **Model 3** | 1.00 (ref) | 1.00 (0.74-1.34) | 0.97 (0.72-1.30) | 0.90 (0.67-1.21) | 0.97 (0.72-1.32) | 0.73 |
| **High-risk prostate cancer** | **Model 1** | 1.00 (ref) | 0.97 (0.65-1.44) | 0.91 (0.60-1.37) | 0.53 (0.34-0.85) | 1.05 (0.69-1.58) | 0.83 |
|  | **Model 2** | 1.00 (ref) | 1.54 (0.87-2.71) | 1.44 (0.81-2.55) | 0.90 (0.50-1.63) | 1.54 (0.87-2.73) | 0.67 |
|  | **Model 3** | 1.00 (ref) | 1.06 (0.73-1.54) | 0.96 (0.66-1.41) | 0.62 (0.41-0.94) | 1.09 (0.74-1.61) | 0.96 |
| **Symptomatic prostate cancer** | **Model 1** | 1.00 (ref) | 0.90 (0.61-1.31) | 1.15 (0.79-1.66) | 0.85 (0.58-1.26) | 1.07 (0.52-1.58) | 0.77 |
|  | **Model 2** | 1.00 (ref) | 1.17 (0.72-1.90) | 1.41 (0.96-2.35) | 1.02 (0.63-1.67) | 1.22 (0.75-1.99) | 0.22 |
|  | **Model 3** | 1.00 (ref) | 0.95 (0.68-1.34) | 1.17 (0.85-1.63) | 0.84 (0.59-1.19) | 1.01 (0.72-1.43) | 0.84 |

**Supplementary Table 3** Background characteristics and dietary intakes of macronutrients and food sources of lignans by quintiles of plasma enterolactone, and tests of their associations, in 1,817 male controls in a nested case-control study within the Malmö Diet and Cancer cohort, 1991-2009

|  | **Q1 (n=357)** | | | **Q2 (n=367)** | **Q3 (n=362)** | | **Q4 (n=379)** | | **Q5 (n=352)** | | **Correlation  (*p* value)^[[5]](#footnote-5)^** | |  |  |  |  |  |
| --- | --- | --- | --- | --- | --- | --- | --- | --- | --- | --- | --- | --- | --- | --- | --- | --- | --- |
| **Median plasma ENL, nmol/L (range)** | **0 (0.0-4.0)** | | | **6.2 (4.1-8.7)** | **11.9 (8.8-15.4)** | | **19.7 (15.5-25.4)** | | **36.0 (25.5-417.2)** | |  | |  |  |  |  |  |
|  |  | | | | | | | | | |  | |  | |  |  |  |
|  | **Mean (SD)** | | | | | | | | | |  | |  | |  |  |  |
| **Age (y)** | 59.0 (5.7) | | | 60.3 (6.3) | 60.5 (6.6) | | 60.5 (6.7) | | 62.5 (7.1) | | 0.15 (<0.001) | |  |  |  |  |  |
| **Height (cm)** | 175.7 (6.5) | | | 176.1 (6.3) | 176.1 (6.4) | | 176.0 (6.4) | | 177.0 (6.5) | | 0.05 (0.024) | |  |  |  |  |  |
| **Weight (cm)** | 83.2 (13.0) | | | 81.8 (12.7) | 81.5 (10.9) | | 79.8 (11.4) | | 81.2 (10.8) | | -0.06 (0.007) | |  |  |  |  |  |
| **BMI (kg/m^2^)** | 26.9 (3.7) | | | 26.4 (3.6) | 26.3 (3.2) | | 25.7 (3.3) | | 25.9 (3.2) | | -0.11 (<0.001) | |  |  |  |  |  |
| **Waist (cm)** | 95.8 (10.9) | | | 94.3 (10.2) | 93.5 (8.9) | | 92.4 (9.7) | | 92.5 (9.4) | | -0.12 (<0.001) | |  |  |  |  |  |
| **Physical activity level (EE/BMR)** | 1.66 (0.42) | | | 1.60 (0.38) | 1.60 (0.37) | | 1.58 (0.35) | | 1.55 (0.31) | | -0.04 (0.055) | |  |  |  |  |  |
|  |  | | | | | | | | | |  | |  | |  |  |  |
|  | **Median (10^th^-90^th^ percentiles)** | | | | | | | | | | |  | |  |  |  |  |
|  |  | |  | | |  | |  | |  | |  | |  |  |  |  |
| **Total energy (kcal)** | | 2550 (1840-3560) | 2520 (1840-3560) | | | 2560 (1780-3480) | | 2450 (1800-3280) | | 2470 (1820-3300) | | -0.08 (0.001) | |  |  |  |  |
| **Fat (E%)** | | 39.6 (32.6-48.6) | 40.5 (32.8-48.7) | | | 40.2 (32.5-47.9) | | 39.2 (31.5-46.9) | | 38.8 (30.7-46.0) | | -0.08 (0.001) | |  |  |  |  |
| **Carbohydrates (E%)** | | 44.2 (36.6-52.1) | 44.3 (36.7-51.3) | | | 44.2 (36.3-52.0) | | 45.0 (37.9-53.0) | | 45.6 (38.8-54.4) | | 0.09 (<0.001) | |  |  |  |  |
| **Protein (E%)** | | 15.5 (12.7-18.9) | 15.2 (12.5-18.7) | | | 15.4 (12.5-18.8) | | 15.5 (12.6-18.7) | | 15.4 (12.6-18.3) | | 0.00 (0.95) | |  |  |  |  |
| **Fibre (g per 1000 kcals)** | | 8.34 (5.61-11.6) | 8.31 (5.63-11.1) | | | 8.41 (5.87-11.9) | | 8.81 (6.26-12.3) | | 9.14 (6.38-13.6) | | 0.15 (<0.001) | |  |  |  |  |
| **High-fibre bread (g)** | | 17.1 (0.0-73.1) | 12.0 (0.0-74.4) | | | 16.2 (0.0-84.7) | | 20.7 (0.0-85.2) | | 20.8(0.0-100.0) | | 0.10 (<0.001) | |  |  |  |  |
| **Vegetables (g)** | | 150 (68-304) | 147 (60-299) | | | 151 (72-287) | | 153 (70-295) | | 150 (72-302) | | 0.03 (0.26) | |  |  |  |  |
| **Fruit and berries (g)** | | 132 (36-293) | 144 (48-335) | | | 143 (38-320) | | 152 (48-332) | | 170 (65-331) | | 0.09 (<0.001) | |  |  |  |  |
| **Fruit juice (g)** | | 0.0 (0.0-179) | 0.0 (0.0-171) | | | 0.0 (0.0-171) | | 0.0 (0.0-186) | | 0.0 (0.0-200) | | 0.02 (0.37) | |  |  |  |  |
| **Coffee (g)** | | 400 (100-1000) | 450 (129-1090) | | | 450 (100-900) | | 400 (100-1000) | | 414 (107-975) | | -0.00 (0.90) | |  |  |  |  |
| **Tea (g)** | | 0.0 (0.0-450) | 0.0 (0.0-450) | | | 0.0 (0.0-386) | | 0.0 (0.0-450) | | 32 (0.0-450) | | 0.06 (0.017) | |  |  |  |  |
| **Wine (g)** | | 0.0 (0.0-143) | 0.0 (0.0-137) | | | 14.3 (0.0-136) | | 0.0 (0.0-143) | | 7.9 (0.0-143) | | 0.02 (0.46) | |  |  |  |  |

Abbreviations: Q, quintile; ENL, enterolactone; SD, standard deviation; BMI, body mass index; EE, calculated energy expenditur; BMR, calculated basal metabolic rate; E%, energy percentage.

**Supplementary Table 4** Background characteristics by quintiles of plasma enterolactone, and tests of association, in 1,817 male controls in a nested case-control study within the Malmö Diet and Cancer cohort, 1991-2009

| **Plasma enterolactone quintile** | **Q1** | **Q2** | **Q3** | **Q4** | **Q5** | **Correlation  (*p* value)^[[6]](#footnote-6)^** |
| --- | --- | --- | --- | --- | --- | --- |
|  |  |  | **N (%)**^[[7]](#footnote-7)^ |  |  |  |
| **Age group** |  |  |  |  |  | 0.12 (<0.001) |
| 46-49 | 27 (8) | 17 (5) | 24 (7) | 26 (7) | 15 (4) |  |
| 50-54 | 65 (18) | 64 (17) | 67 (19) | 60 (16) | 48 (14) |  |
| 55-59 | 103 (29) | 86 (23) | 74 (20) | 82 (22) | 64 (18) |  |
| 60-64 | 107 (30) | 114 (31) | 101 (28) | 115 (30) | 85 (24) |  |
| 65-69 | 44 (12) | 59 (16) | 60 (17) | 54 (14) | 56 (16) |  |
| 70-73 | 11 (3) | 27 (7) | 36 (10) | 42 (11) | 84 (24) |  |
|  |  |  |  |  |  |  |
| **Season** |  |  |  |  |  |  |
| Winter | 87 (24) | 98 (27) | 94 (26) | 108 (28) | 95 (27) | (0.050) |
| Spring | 93 (26) | 102 (28) | 102 (28) | 114 (30) | 126 (36) |  |
| Summer | 59 (17) | 63 (17) | 52 (14) | 43 (11) | 35 (10) |  |
| Fall | 118 (33) | 104 (28) | 114 (31) | 114 (30) | 96 (27) |  |
| **Educational status** |  |  |  |  |  | 0.05 (0.013) |
| Elementary | 183 (51) | 179 (49) | 166 (46) | 177 (47) | 143 (41) |  |
| Primary and secondary | 71 (20) | 64 (18) | 72 (20) | 74 (20) | 87 (25) |  |
| Upper secondary | 34 (10) | 38 (10) | 42 (12) | 45 (12) | 32 (9) |  |
| Further education, university degree | 68 (19) | 84 (23) | 82 (23) | 81 (21) | 89 (25) |  |
| **Smoking status** |  |  |  |  |  | -0.11 (<0.001) |
| Never-smokers | 80 (22) | 87 (24) | 117 (32) | 122 (32) | 118 (34) |  |
| Former smokers | 158 (44) | 162 (44) | 147 (41) | 167 (44) | 166 (47) |  |
| Current smokers | 119 (33) | 117 (32) | 96 (27) | 90 (24) | 68 (19) |  |
| **Alcohol habits** |  |  |  |  |  | -0.02 (0.40) |
| Zero consumers | 29 (8) | 36 (10) | 25 (7) | 24 (6) | 20 (6) |  |
| < 20 g alcohol per day | 217 (61) | 240 (65) | 234 (65) | 264 (70) | 246 (70) |  |
| 20-40 g alcohol per day | 85 (24) | 69 (19) | 72 (20) | 70 (18) | 66 (19) |  |
| > 40 g alcohol per day | 26 (7) | 22 (6) | 31 (9) | 21 (6) | 20 (6) |  |
| **Physical activity level** |  |  |  |  |  | -0.03 (0.11) |
| Quartile 1 | 95 (27) | 91 (25) | 86 (24) | 98 (26) | 85 (24) |  |
| Quartile 2 | 76 (21) | 87 (24) | 99 (27) | 106 (28) | 85 (24) |  |
| Quartile 3 | 65 (18) | 98 (27) | 90 (25) | 73 (19) | 115 (33) |  |
| Quartile 4 | 121 (34) | 91 (25) | 87 (24) | 102 (27) | 67 (19) |  |
| **Prevalent diabetes** |  |  |  |  |  | (0.59) |
| No | 344 (96) | 355 (97) | 345 (95) | 359 (95) | 334 (95) |  |
| Yes | 13 (4) | 12 (3) | 17 (5) | 20 (5) | 18 (5) |  |
| **Current antibiotic use** |  |  |  |  |  | (0.73) |
| No | 353 (99) | 364 (99) | 357 (99) | 377 (99) | 347 (99) |  |
| Yes | 4 (1) | 3 (1) | 5 (1) | 2 (1) | 5 (1) |  |
| **Energy reporting** |  |  |  |  |  | (0.008) |
| Under | 64 (18) | 40 (11) | 48 (13) | 40 (11) | 33 (9) |  |
| Adequate | 283 (79) | 305 (83) | 299 (83) | 326 (86) | 307 (87) |  |
| Over | 10 (3) | 22 (6) | 15 (4) | 13 (3) | 12 (3) |  |
| **Changed diet** |  |  |  |  |  | (0.002) |
| No | 286 (80) | 306 (83) | 291 (81) | 291 (77) | 253 (72) |  |
| Yes | 71 (20) | 61 (17) | 69 (19) | 88 (23) | 99 (28) |  |

**Supplementary Table 5** Odds ratios (with 95 % confidence intervals within parentheses) for prostate cancer by quintiles of plasma enterolactone concentration in men by age below or over the age of 70 in a nested case-control study within the Malmö Diet and Cancer cohort, 1991-2009

|  | **No. events** | **Age** | |  |
| --- | --- | --- | --- | --- |
|  |  | **<70 y (n=2496)** | **≥70 y (n=319)** |  |
| Total prostate cancer |  |  |  |  |
| Q1 | 191/10 | 1.00 (ref) | 1.00 (ref) |  |
| Q2 | 188/23 | 0.99 (0.77-1.28) | 1.03 (0.36-2.91) |  |
| Q3 | 183/21 | 0.98 (0.76-1.27) | 0.68 (0.24-1.90) |  |
| Q4 | 153/27 | 0.78 (0.60-1.02) | 0.75 (0.28-2.05) |  |
| Q5 | 172/40 | 1.09 (0.83-1.42) | 0.53 (0.20-1.37) |  |
| *P* for trend |  | 0.86 | 0.059 |  |
| *P* for interaction |  | 0.063 | |  |
| Low-risk prostate cancer |  |  |  |  |
| Q1 | 123/5 | 1.00 (ref) | 1.00 (ref) |  |
| Q2 | 125/6 | 1.01 (0.76-1.36) | 0.60 (0.14-2.67) |  |
| Q3 | 118/12 | 0.97 (0.72-1.30) | 0.78 (0.21-2.86) |  |
| Q4 | 111/19 | 0.87 (0.64-1.19) | 1.16 (0.34-3.98) |  |
| Q5 | 110/18 | 1.06 (0.77-1.45) | 0.44 (0.13-1.48) |  |
| *P* for trend |  | 0.79 | 0.16 |  |
| *P* for interaction |  | 0.20 | |  |
| High-risk prostate cancer |  |  |  |  |
| Q1 | 66/5 | 1.00 (ref) | 1.00 (ref) |  |
| Q2 | 61/16 | 0.94 (0.64-1.38) | 1.23 (0.35-4.39) |  |
| Q3 | 64/9 | 1.00 (0.68-1.46) | 0.52 (0.14-1.95) |  |
| Q4 | 40/8 | 0.60 (0.39-0.92) | 0.38 (0.10-1.47) |  |
| Q5 | 61/22 | 1.12 (0.75-1.67) | 0.54 (0.16-1.81) |  |
| *P* for trend |  | 0.91 | 0.14 |  |
| *P* for interaction |  | 0.16 | |  |
| Symptomatic prostate cancer |  |  |  |  |
| Q1 | 81/6 | 1.00 (ref) | 1.00 (ref) |  |
| Q2 | 77/11 | 0.94 (0.66-1.34) | 0.79 (0.23-2.77) |  |
| Q3 | 95/16 | 1.18 (0.84-1.65) | 0.88 (0.27-2.88) |  |
| Q4 | 73/9 | 0.87 (0.61-1.24) | 0.41 (0.12-1.46) |  |
| Q5 | 77/18 | 1.12 (0.78-1.61) | 0.39 (0.13-1.25) |  |
| *P* for trend |  | 0.66 | 0.027 |  |
| *P* for interaction |  | 0.023 | |  |

Unconditional logistic regression; models adjusted for age, baseline date, height, waist circumference, education, smoking habits and season.

Abbreviation: Q, quintile.

1. Unconditional logistic regression model adjusted for age, baseline date, season, height (continuous), waist circumference (continuous), educational level (categorical), and smoking status (categorical). [↑](#footnote-ref-1)
2. Excluding participants who had changed their diets significantly, see text. [↑](#footnote-ref-2)
3. Excluding participants with plasma enterolactone below the detection limit, see text. [↑](#footnote-ref-3)
4. Excluding participants with follow-up time of less than 2 years (n=68). [↑](#footnote-ref-4)
5. Spearman’s rho and tests for associations. [↑](#footnote-ref-5)
6. Kendall’s tau-b (ordinal variables) and χ^2^ tests (nominal variables). [↑](#footnote-ref-6)
7. Percentages do not always add up to 100 because of rounding. [↑](#footnote-ref-7)
