# Supplementary material for: Plasma enterolactone and risk of prostate cancer in middle-aged Swedish men
Source: Eur J Nutr. 2017 Sep 7;57(7):2595–606. doi: 10.1007/s00394-017-1530-z (PMC6182673; doi:10.1007/s00394-017-1530-z)
Supplement: Supplementary file 2 — Supplementary material 2 (DOC 31 kb) [file 394_2017_1530_MOESM2_ESM.doc]

**Supplementary Figure 1** Restricted cubic spline regression of plasma enterolactone and odds ratios of high-risk prostate cancer with 95 % confidence intervals in a nested case-control study within the Malmö Diet and Cancer cohort, 1991-2009

Note: Enterolactone distribution truncated at 100 nmol/L
